# Efficacy of a single oral administration of a formulation of fluralaner, moxidectin and pyrantel (BRAVECTO® TriUNO) in dogs for the treatment and prevention of angiostrongylosis

**DOI:** 10.1186/s13071-026-07529-4

**Published:** 2026-07-24

**Authors:** Nadja Rohdich, Rafael Chiummo, Eva Zschiesche, Marie-Kristin Raulf, Anna Schwarz, Anne McLoughlin, Manuela Schnyder, Jenny Schulte Bocholt, Kristina Merhof, Manon Mikić, Christina Strube, Lea Heinau

**Affiliations:** 1 MSD Animal Health Innovation GmbH, Schwabenheim an der Selz, Germany; 2 MSD Saúde Animal, São Paulo, Brazil; 3 Institute for Parasitology, University of Veterinary Medicine Hannover, Hannover, Germany; 4 Present Address: Institute for Animal Hygiene, Animal Welfare and Farm Animal Behaviour, University of Veterinary Medicine Hannover, Hannover, Germany; 5 Iorras Product Development, Glenamoy–Ballina, Ireland; 6 Institute of Parasitology, Vetsuisse Faculty, University of Zurich, Zurich, Switzerland; 7 Small Animal Clinic, University of Veterinary Medicine Hannover, Hannover, Germany

**Keywords:** *Angiostrongylus vasorum*, Bravecto, Canine, Fluralaner, Lungworm, Milbemycin, Moxidectin, Pyrantel

## Abstract

**Background:**

Four studies investigated the efficacy of fluralaner/moxidectin/pyrantel chewable tablets (Bravecto® TriUNO) in preventing and treating canine angiostrongylosis; one study also included a formulation of fluralaner/milbemycin oxime.

**Methods:**

Studies included eight or 10 dogs/group. In Studies 1 and 2 (prevention), dogs inoculated with third-stage larvae of *Angiostrongylus vasorum* on day − 31 or − 28 were randomized to groups by sex and body weight; in Studies 3 and 4 (treatment) by faecal first-stage larvae counts at approximately 8 weeks post-inoculation (PI). All studies included an untreated control group (CG). Study 1 included three groups treated with formulations of moxidectin/fluralaner/pyrantel; minimum moxidectin doses 0.0125, 0.025 or 0.055 mg/kg. The formulation with moxidectin at 0.025 mg/kg, 99.0% effective in preventing establishment of infection, was adopted as the investigational veterinary product (IVP) for the following studies. Study 2 included a group treated with either the IVP or a combination of fluralaner (10 mg/kg) with milbemycin oxime (0.75 mg/kg) (IVP-2). In all studies, the IVP was administered once on day 0; in Study 2, IVP-2 was administered on days 0, 31, 62 and 93. In Study 1, efficacy was determined by reductions in geometric mean necropsy worm counts approximately 33 days post-treatment versus mean CG counts, in the other studies by reductions in mean faecal L1 counts. Study 2 assessed pulmonary changes via thoracic computed tomography. Respiratory signs and serological antibody responses were monitored.

**Results:**

In Study 1, all moxidectin doses exceeded 90% efficacy. In Study 2, IVP efficacy (one treatment) at 62 days was 100.0%; IVP-2 (four treatments) efficacy was 99.8% at 124 days. Respiratory signs were absent or lower in IVP-treated than CG dogs. In Studies 3 and 4, IVP efficacy was 100% by 3 and 4 weeks post-treatment, respectively. In all studies, lungworm count reductions in the IVP groups were statistically significant (*P* < 0.0001) versus the CG. Serology confirmed *A. vasorum* infections in all control and IVP-2-treated dogs, and absence of infections in IVP-treated dogs. Treatments were well tolerated.

**Conclusions:**

A single treatment with Bravecto TriUNO is highly effective in preventing angiostrongylosis and eliminating established *A. vasorum* infections. Infection-related lung pathology was reduced in treated dogs.

**Graphical abstract:**

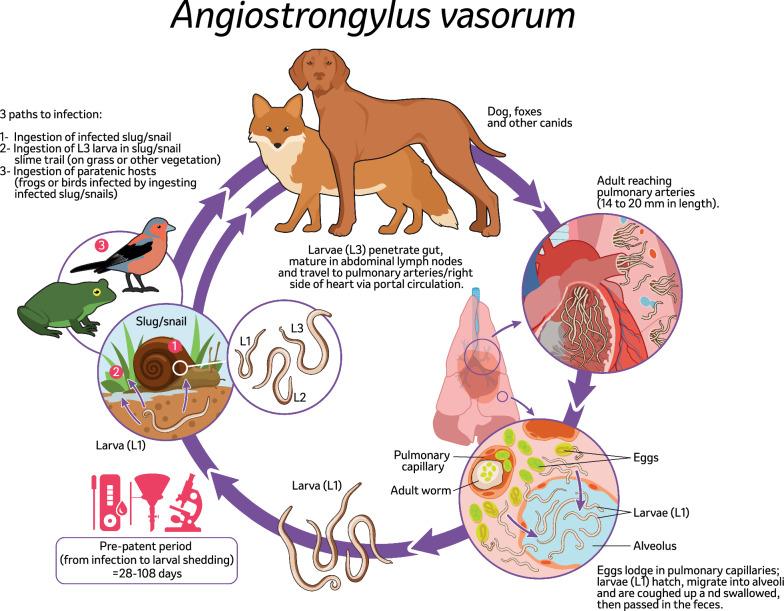

**Supplementary Information:**

The online version contains supplementary material available at 10.1186/s13071-026-07529-4.

## Background

The metastrongyloid nematode *Angiostrongylus vasorum* is a parasite of domestic dogs and wild canid species. Infections may remain subclinical or result in non-specific clinical signs that can lead to difficulties in diagnosis. The most commonly reported manifestations are respiratory in nature, arising from cardio/pulmonary effects [1, 2]. Gastrointestinal signs are less commonly reported, and infections have also resulted in coagulopathies and neurologic disturbances along with other organ system pathologies. In severe cases the outcome of infection with *A. vasorum* may be fatal [1, 3, 4]. Reports of the occurrence of canine angiostrongylosis indicate a geographic expansion within and beyond countries throughout Europe, from the United Kingdom eastward to Romania, [1, 3–7]. *Angiostrongylus vasorum* has also been reported in areas of North and South America and Africa [8–12].

Adult *A. vasorum* inhabit the heart and pulmonary arteries of infected canids, releasing eggs that pass to the lung capillaries where first-stage larvae (L1) develop [2, 4]. The L1 migrate to the alveoli and up the respiratory tree to be coughed up, swallowed and passed in faeces in high numbers (as many as 17,000 per gramme), although there may be large daily variation in larval shedding [1, 2, 4]. Within intermediate hosts (mainly gastropods, sometimes amphibians), development occurs to infective third-stage larvae (L3) [13]. Canids are infected by ingestion of intermediate or paratenic hosts or by directly consuming L3 from the environment [1, 4]. Once ingested by a canid, the larvae penetrate the gut wall, migrate to abdominal lymph nodes and moult to the fourth stage (L4) to then be carried in the blood to the pulmonary arteries. Within an infected definitive canid host the prepatent period of *A. vasorum* is reported to be approximately 5–7 weeks [2, 4].

Reported and labelled options for the treatment of canine *A. vasorum* infections include different formulations of the macrocyclic lactones moxidectin and milbemycin oxime. Both are long-established as being safe and effective in the treatment of canine intestinal parasite infections and in the prevention of heartworm disease caused by *Dirofilaria immitis* [4, 14]. A moxidectin formulation at 0.024 mg/kg (in combination with sarolaner and pyrantel) was shown to be effective against immature adult stages of *A. vasorum* infections administered orally on a single occasion [15]. Another moxidectin combination product used for treatment of patent infections with *A. vasorum* is a topical formulation used at 2.5 mg/kg body weight [16, 17]. In individual cases up to three administrations at 4 weekly intervals were needed to resolve the infection [16]. Milbemycin oxime is administered orally, at 0.5 mg/kg at weekly intervals for four weeks to treat angiostrongylosis or at monthly intervals in combination with afoxolaner or praziquantel to prevent the disease [8, 18, 19]. Off-label oral fenbendazole, administered daily for three weeks, has also proven to be effective [4, 17].

Novel combination chewable tablet formulations of fluralaner were recently developed for broad spectrum internal and external parasite control in dogs; one containing 12.5% (w/w) fluralaner, 0.03% (w/w) moxidectin and 6.25% (w/w) pyrantel (the investigational veterinary product, IVP), the other 5.00% (w/w) fluralaner with 0.375% (w/w) milbemycin oxime (IVP-2). Here we report on four laboratory studies—two were conducted to evaluate the efficacy of the IVP in preventing canine angiostrongylosis (whereby one study included an IVP-2 group), and two were conducted to confirm the efficacy of the IVP in treating infections with *A. vasorum*.

## Methods

Each study was single-site, investigator-masked, negative-controlled, randomized, and completed in alignment with “Good clinical practice” VICH GL9 (GCP) (CVMP/VICH/595/98-Final), Efficacy of anthelminthics—General requirements” VICH GL7 (CVMP/VICH/832/99-corr), and “WAAVP guidelines for evaluating the efficacy of anthelmintics for dogs and cats” [20]. Notably, in consideration of the WAAVP guidelines encouraging the use of in vivo diagnosis of nematode infections, three of these studies implemented a non-terminal protocol. The protocol for each study was reviewed and approved by the ethics committee of the contract facilities undertaking the studies, by the relevant authorities in Ireland and the ethics commission of the Animal Care and Use Committee of the German Lower Saxony State Office for Consumer Protection and Food Safety (Niedersaechsisches Landesamt fuer Verbraucherschutz und Lebensmittelsicherheit) and by the ethics committee of MSD Animal Health Innovation GmbH.

An outline of the details of each study is provided in Table 1.

**Table 1:** Summary of the designs of studies to determine efficacy in the prevention of angiostrongylosis and treatment of *A. vasorum* infections

	Study 1 (prevention)		Study 2 (prevention)	Study 3 (treatment)		Study 4 (treatment)
Number of dogs per group	8		10	8		10
Day *A. vasorum* larvae inoculated (number of larvae)	− 31 (200)		− 28 (250)	− 60		− 56
Randomized by	Sex, body weight			Faecal larval counts	Sex, faecal larval counts	
Study groups	Fluralaner/moxidectin/pyrantel^a^		1. Fluralaner/moxidectin^b^/pyrantel	1. Fluralaner/moxidectin^b^/pyrantel		
	1. Moxidectin	0.0125 mg/kg	2. Fluralaner/milbemycin oxime^c^	2. Control group (untreated)		
	2. Moxidectin	0.0250 mg/kg^b^	3. Control group (untreated)			
	3. Moxidectin	0.050 mg/kg				
	4. Control group (untreated)					
Day(s) of treatment	Day 0		1. Day 0 2. Days 0, 31, 62, 93	Day 0		Day 0
Post-treatment assessment						
Faecal samples	Weekly		3 times weekly	3 times weekly		3 times weekly
Blood samples	Weekly		Weekly	Weekly		Weekly
Respiratory			Twice weekly	Twice weekly		Weekly
Thoracic computed tomography			Between days 54–61, 117–124			
Animal phase completion	Days 33–35 (necropsy)		1. Day 69 2. Day 122	Day 28		Day 28

^a^Fluralaner doses (minimum) administered in each formulation: 1. 10.0 mg/kg; 2. 20.0 mg/kg; 3. 10.0 mg/kg

Pyrantel doses (minimum) administered in each formulation: 1. 2.5 mg/kg; 2. 5.0 mg/kg; 3. 10.0 mg/kg

^b^Formulation (moxidectin dose) adopted for following studies

^c^Fluralaner dose 10 mg/kg, milbemycin oxime 0.75 mg/kg

### Animals and management

Study dogs were purpose-bred Beagles, male and female, with an age range of 8.4–37 months. For inclusion, dogs were required to: not have been treated with any topical or systemic antiparasitic product for at least 2 months preceding the beginning of acclimatization; have a suitable temperament to allow study assessments; not be pregnant; have had negative faecal examinations for *A. vasorum* L1 at least twice prior to *A. vasorum* inoculation; and to have been *A. vasorum* antigen and antibody negative from testing of pre-study blood samples collected early in the acclimatization phase.

During acclimatization, inoculation and treatment phases of each study, dogs were pair-housed within group and sex in pens meeting animal welfare requirements compliant with EU Directive 63/2010. Exceptions for pair housing occurred on the days of treatment and inoculation of *A. vasorum* L3, and in Study 2 on days of computed tomography: at those times, dogs were individually housed. Dogs were also single-housed on days of faeces collections. According to the group housing arrangements, dogs had daily access to an exercise area, with only few exceptions (e.g. on days when anaesthetized for L3 inoculations and computed tomography procedures). No contact was possible between dogs from different treatment groups. All were fed an age-appropriate commercial diet, and water was available ad libitum*.* On the day of anthelmintic treatments, dogs were offered half of their daily ration up to 20 min prior to treatment, taking care that an adequate amount of food had been ingested.

### Diagnostic procedures and clinical assessments

Faecal samples were examined for the presence of *A. vasorum* L1 using the Baermann technique [21]. Differences in respiratory parameters were based on assessments of: respiratory frequency, intensity of inspiratory and expiratory sounds on a 4-point scale (0 = no sound; 1 = slight; 2 = moderate; 3 = severe); quality of sound (normal/deepened/stertor/stridor/rhonchus/wheeze/crackle); abdominal involvement; panting; and cough/retch (yes or no). Blood samples were examined for circulating antigens using a sandwich-ELISA based on monoclonal and polyclonal antibodies directed against adult *A. vasorum* excretory/secretory (E/S)–antigen (sensitivity 95.7%, specificity 94.0%) [22]. Blood samples were also tested for specific antibodies with an ELISA using *A. vasorum* adult E/S antigen and monoclonal antibodies (sensitivity 85.7% specificity of 98.8%) [23]. Health observations were completed daily, and body weights were collected at regular intervals. Immediately and up to 8 h following treatment, and daily throughout the study, dogs were observed for any adverse events.

### Inoculations with *Angiostrongylus vasorum*

The *A. vasorum* L3 used for inoculations were harvested from experimentally infected *Helix aspersa* /*Achatina fulica* snails maintained at each research facility. In one prevention study (Study 1) and one treatment study (Study 3) the *A. vasorum* isolate was of Swiss origin, collected from a dog in October 2019. For studies 2 and 4, the isolates were collected from a dog in Denmark in 2012, and from a dog in Hannover, Germany in 2021, respectively. Inoculation was completed in dogs anaesthetized with either propofol or an acepromazine/buprenorphine/propofol protocol. Approximately 200 to 250 *A. vasorum* L3 were inoculated intragastrically using a gavage tube that was then flushed to ensure the full larval dose had been dispensed. Inoculation was immediately followed by anti-emetic treatment (maropitant or metoclopramide).

### Studies 1 and 2—preventing angiostrongylosis

The objective of these studies was to determine the most appropriate dose of the IVP chewable tablet formulation (Study 1) and then confirm the efficacy of that dose for the prevention of angiostrongylosis in dogs. In the post-treatment period of both studies, blood and faecal samples were collected at least once weekly, and respiratory assessments were recorded twice weekly.

#### Study 1

This dose-finding study assessed the efficacy of a single administration of three dosages of the formulation, with the primary efficacy objective based on mean reductions in adult worm counts recovered from dogs in treated and control groups at necropsy. Thirty-two dogs (16 female, 16 male) were ranked within sex by decreasing body weights into eight blocks of four and, using random order numbers from Fisher and Yates tables, allocated to be treated with the investigational formulations, including moxidectin, fluralaner and pyrantel, or to be included in a sham-treated control group (CG). Dogs randomized to active treatment groups received minimum moxidectin doses of 0.0125, 0.025 or 0.050 mg/kg body weight on day 0. Minimum fluralaner doses in the respective formulations were 5, 10 and 20 mg/kg, and pyrantel 2.5, 5.0 and 10.0 mg/kg. Inoculation of *A. vasorum* L3 was completed on day − 31.

Study dogs were humanely euthanized on either day 33, 34 or 35 in multiples of four (one dog from each group). The thorax of each dog was opened and reverse lung perfusion performed. The aorta and *venae cavae caudalis* and *cranialis* were clamped and a plastic tube placed into the *truncus pulmonalis* to collect outflowing blood. Using a probe inserted into the left ventricle, approximately 2 L of isotonic saline (~ 37 ± 2 °C) were pumped through pulmonary veins, lung capillaries and pulmonary arteries to the pulmonary trunk. The perfusion solution and blood were collected from the *truncus pulmonalis,* and poured onto a fine sieve (≤ 100 μm mesh). The sieve contents were rinsed with saline into a container and examined utilizing a dissecting microscope. The viability of the isolated worms was determined by observing any movement. For fragments, the total of heads and female and male tails was counted. Whole worms and fragments were counted and sexed (except head fragments).

#### Study 2

This dose confirmation study also included an IVP-2 group (milbemycin oxime 0.75 mg/kg, fluralaner 10 mg/kg). The primary efficacy objective was based on post-treatment reductions in faecal L1 counts in dogs inoculated with L3 on day − 28. On day − 4, 30 dogs were ranked by sex and descending body weights, placed into ten blocks of three and within blocks randomized among three treatment groups using the Microsoft Excel (Microsoft Corporation, Redmond, USA) random number generator. Dogs in the CG were sham-treated. Dogs in the second group were treated once, on day 0, with the IVP containing moxidectin at the 0.025 mg/kg dose selected from Study 1. The third group (IVP-2) was treated orally on days 0, 31, 62 and 93. This dose frequency and study duration was based on an earlier 92-day study investigating the efficacy against *A. vasorum* of a marketed formulation of afoxolaner combined with milbemycin oxime (dose 0.5 mg/kg) administered on days 0, 28, 56 and 84 [18]. At that dose frequency and lower dose than in this study, efficacy against adult *A. vasorum* was 94.9% [18]. Therefore, while post-treatment observations in the current study concluded on day 62 for the IVP group, for the CG and IVP-2 group, observations continued until day 124. Faecal L1 count comparisons with the CG were assessed for samples from days 28 to 62 in the IVP group, and days 93 to 124 in the IVP-2 group.

Thoracic computed tomography (CT) scans of study dogs were performed using a spectral-detector CT Scanner (Philips IQon Spectral CT, Philips Healthcare Germany) between days − 35 to − 33, − 7 to – 1 and 54 to 61, for all animals in all groups, and on days 117 to 124 for all animals of the CG and IVP-2 groups. Similar ratios of the study groups were scanned on each day. All CT scans were conducted under general anaesthesia and performed in sternal recumbency. All dogs were premedicated with dexmedetomidine (4 μg/kg) and methadone (0.3 mg/kg) intramuscularly; anaesthesia was induced with propofol titrated by effect intravenously and maintained after intubation with isoflurane in an oxygen–air mixture. The dogs were ventilated with the following ventilation protocol: volume-controlled ventilation with a tidal volume (VT) of 12 mL/kg in relation to the initial body weight at the first CT time point, PEEP of 3 cmH_2_O, FiO_2_ 0.5, inspiratory pause of 20%, RR/min selected so that normocapnia was achieved. A native scan was performed, followed by a venous phase with the following parameters: 140 kV maximum tube potential, automatic mAs, and a pitch of 0.6, with a gantry rotation time of 1 s, a slice thickness of 1 mm and a 512 image matrix. Each patient received 700 mg iobitridol per kg bodyweight (2 mL/kg of Xenetix 350, Guerbet GMBH, Sulzbach, Germany) intravenously administered via a power dual injector system (MEDRAD, Stellant/Bayer HealthCare, Leverkusen, Germany. The images were evaluated in a lung window (window level: −600 Hounsfield units; window width: 1500 Hounsfield units) and a soft tissue window (window level: 30 Hounsfield Units; window width 350 Hounsfield Units). The presence of the following parameters was assessed for each lung lobe: periarterial, hyperattenuating interstitial infiltrates; increased attenuation; bronchial wall thickening and pleural effusion. Additionally, the size, course, lumen and vessel wall thickness of the pulmonary arteries, the heart, pleura, and intrathoracic lymph nodes were evaluated for any developing pathology.

### Studies 3 and 4—treating *Angiostrongylus vasorum* infection

The objective of these studies was to confirm the selected dose of the IVP for treating dogs with established *A. vasorum* infections. Dogs were inoculated once with *A. vasorum* L3 on either day −60 (Study 3) or −56 (Study 4). For any dog to continue in the study, infection had to be confirmed by a positive faecal test for *A. vasorum* L1 from multiple samples collected from day − 8 to day − 1. On day − 1, eight and 10 pairs of dogs in studies 3 and 4, respectively, were ranked by descending minimum larval count (within sex in Study 4) and randomly allocated to study groups in blocks of two using the Microsoft Excel (Microsoft Corporation, Redmond, USA) random number generator. On day 0, Group 1 received the IVP once, and Group 2 remained unmedicated, and CG dogs were either sham-treated or mock-dosed with water. Faeces were collected three times weekly until study termination on day 28. Blood was sampled at weekly intervals to determine *A. vasorum* antigen and antibody optical density (OD) values. Respiratory assessments were performed at the start of acclimatization and then twice weekly from day 7 throughout study 3 and once weekly in study 4.

#### Statistics

In each study, the experimental and statistical unit was the individual dog, and the primary efficacy objectives were based on geometric means. Statistical analyses were performed with the software package SAS® (SAS Institute Inc., Cary, NC, USA, release 9.4).

In Study 1, the mean number of worms (intact and head of worm fragments) was compared between the treated groups and the untreated CG using Abbott’s formula:

$${\text{Efficacy }}\% \frac{{{\overline{\mathrm{X}}}_{{{\mathrm{CG}}}} - {\overline{\mathrm{X}}}_{{{\mathrm{Treated}}}} }}{{{\overline{\mathrm{X}}}_{{{\mathrm{CG}}}} }} * 100,$$

where ${\overline{\mathrm{X}}}_{{{\mathrm{CG}}}}$ is the mean number of worms in the CG and ${\overline{\mathrm{X}}}_{{{\mathrm{Treated}}}}$ the mean worm number in the respective treated group.

Necropsy worm counts in the treated and control groups were compared pairwise using a linear mixed model with fixed factor of the study group. Randomization block was included as a random effect. Pairwise comparisons of treated versus control groups were performed using two-tailed tests with the level of significance set to *α* = 0.05. Worm counts were log-transformed including a shift of 1 prior to the analysis to allow calculation due to zero counts: *x*’ = loge (*x* + 1). Treatment was declared efficacious if the control group was adequately infected, i.e. ≥ 5 worms were recovered at necropsy from ≥ 6 CG dogs, if geometric mean counts in treated groups, compared with the CG, were significantly different (*P* ≤ 0.05), and if worm counts were reduced by ≥ 90%.

In Study 2, efficacy evaluations were considered valid if L1 were recovered from the faeces of all CG dogs in the collection periods from day 28 to day 62 (for comparison with the IVP group) and from days 93 to 124 (for comparison with the IVP-2 group). In Studies 3 and 4, evaluations were considered valid if L1 were recovered in faecal samples from all dogs prior to treatment, and from ≥ 6 CG dogs at post-treatment collections completed at least 3 times per week through day 28. Faecal larval counts were summarized using individual maxima per week, and geometric and arithmetic weekly group means of the individual maxima were calculated. In studies 2, 3 and 4, reductions of L1 counts of treated versus control groups were calculated with the geometric and arithmetic means of the 90th percentile of individual counts using Abbott’s formula:

$${\mathrm{Reduction}}\left( \% \right) = \frac{{{\mathrm{Fecal}}\;{\text{ larval}}\;{\text{ count}}_{{({\mathrm{CG}})}} - {\text{ Fecal}}\;{\text{ larval}}\;{\text{ count}}_{{({\mathrm{IVP}})}} }}{{{\mathrm{Fecal}}\;{\text{ larval}}\;{\text{ count}}_{{({\mathrm{CG}})}} }} \times 100,$$

where CG is the mean (geometric and arithmetic) of the control group faecal larval counts and IVP is the mean of the IVP and IVP-2 treated groups.

The 90th percentile was selected to represent individual faecal L1 counts to obtain a robust measure for larval excretion that follows the worst-case principle, but was less biased by possible outliers than the individual maximum. The validity of the results was confirmed by a pairwise statistical comparison of the maximum individual counts in the treated group to the CG using a linear mixed model, including study group as a fixed effect and randomization block as a random effect, with the level of significance set to *α* = 0.05 (two-sided). Individual maxima were log-transformed and shifted prior to the statistical analysis: *x*’ = loge (*x* + 1). Adequacy of infection in Study 2 was determined in the CG after day 0. In Studies 3 and 4 the efficacy determination, calculated using Abbott’s formula, required an adequate infection in both study groups pre-treatment and in the CG post-treatment. In all studies treatment was deemed effective if a reduction of maximum faecal L1 counts ≥ 90% was achieved and if the mean maximum faecal larval counts in the treated versus control groups were significantly different (*P* ≤ 0.05).

For secondary efficacy criteria, arithmetic means of respiratory parameters and antigen and antibody ELISA results were summarized for each group by day of collection.

## Results

In all studies groups were well matched by sex and body weight. All blood and faecal samples at the beginning of acclimatization established that dogs had not been previously exposed to *A. vasorum*. In each study, the results of faecal analysis and of antigen and antibody tests confirmed the presence of adult *A. vasorum* with larval excretion in all CG dogs.

### Studies 1 and 2—preventing angiostrongylosis

#### Study 1—preventive efficacy (Table 2)

**Table 2:** Study 1, counts of adult *Angiostrongylus vasorum* recovered at necropsy (eight dogs/group) on either day 33, 34 or 35

	Control group (CG) (Sham-treated)	Fluralaner 5 mg/kg Moxidectin 0.0125 mg/kg Pyrantel 2.5 mg/kg	Fluralaner 10 mg/kg Moxidectin 0.025 mg/kg Pyrantel 5 mg/kg	Fluralaner 20 mg/kg Moxidectin 0.05 mg/kg Pyrantel 10 mg/kg
GM (AM)	78.0 (85.0)	6.1 (8.4)	0.8 (1.1)	0.0 (0.0)
Median	87.5	6.0	1.0	0.0
Minimum, maximum	32, 137	1, 21	0, 3	0, 0
Efficacy % GM (AM)		92.2 (90.1)	99.0 (98.7)	100.0 (100.0)
Statistics (comparison versus untreated)				
*F*-statistic		71.34	175.06	233.38
*P*-value		< 0.0001	< 0.0001	< 0.0001

^*^Degrees of freedom (DF) of the statistical tests were 1 (numerator DF) and 17 (denominator DF) in each post-treatment week

GM: geometric mean; AM: arithmetic mean

Inoculation with *A. vasorum* third-stage larvae on day − 31; treatments on day 0

The total of intact worms and heads of worm fragments was at least five in all eight CG dogs, confirming the adequacy of infection to provide efficacy estimates of the moxidectin doses in preventing establishment of infection with *A. vasorum*. For the primary objective, the efficacy of all doses exceeded 90%, and efficacy of the two higher doses was ≥ 99% (Table 2). Mean worm counts in all groups that received any moxidectin dose were significantly different from the CG (*P* < 0.0001).

Following inoculation of *A. vasorum* L3 on day − 31, L1 were first detected in a faecal sample from one CG dog on day 14. Samples from all dogs in this group were positive on day 26, one dog was again negative on days 27 and 28, and then positive at the final sampling on day 33. Arithmetic mean larval counts from CG faecal samples increased progressively from day 14 (mean 0.1) through day 33 (672.0). In the group that received the lowest dose of moxidectin, the only positive finding of *A. vasorum* L1 was from a dog sampled on day 19. In the middle- and highest-dose groups, the only positive findings of faecal LI were on day 33 when arithmetic mean counts were 0.3 and 0.1, respectively.

Mean respiratory rates increased from larval inoculation on day − 31 to day − 1 for all study groups. In the IVP-treated groups there were fluctuations in respiratory rates throughout the sampling period, with no remarkable changes. From the observations on day 8 through day 33 the respiratory rate in the CG was higher than in the IVP-treated groups (Additional file 1: Fig. S1).

**Fig. 1:**
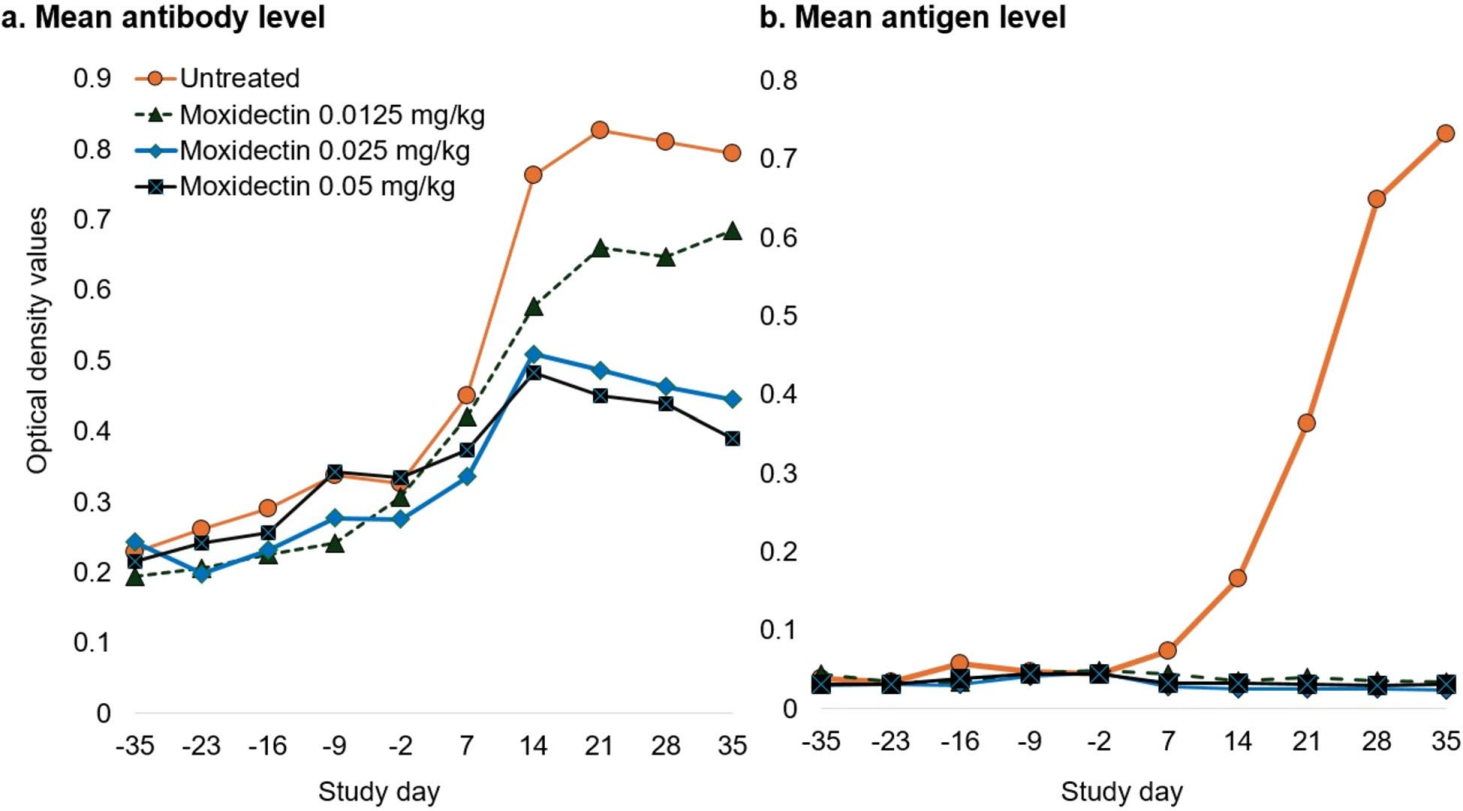
Study 1: mean antibody **a**. and antigen **b**. levels in treated and sham-treated control groups. *Angiostrongylus vasorum* third-stage larvae inoculated on day − 31. Treated groups received moxidectin (doses in mg/kg) in formulations also containing fluralaner and pyrantel. Treatments were administered on day 0 (8 dogs per group)

Mean antibody levels increased in all groups between day − 2 and day 14. In the CG and in the lowest-dose group the mean antibody levels continued to increase, with occasional slight declines from the previous sampling, until the day of necropsy. In the two higher-dose groups, there was a progressive decline in antibody levels from day 21 to day 35 (Fig. 1a). Mean antigen levels in the treated groups remained at or close to baseline throughout the study, while levels in the CG increased markedly from day 7 through the final sampling on day 35 (Fig. 1b).

#### Study 2—preventive efficacy

Three dogs in the CG were withdrawn on day 44 after prolonged inappetence and weight loss, likely caused by the *A. vasorum* infection. Another dog in this group was removed after collapsing during a CT procedure. All four dogs were successfully treated and had a full recovery.

### Faecal larval counts (Fig. 2)

**Fig. 2:**
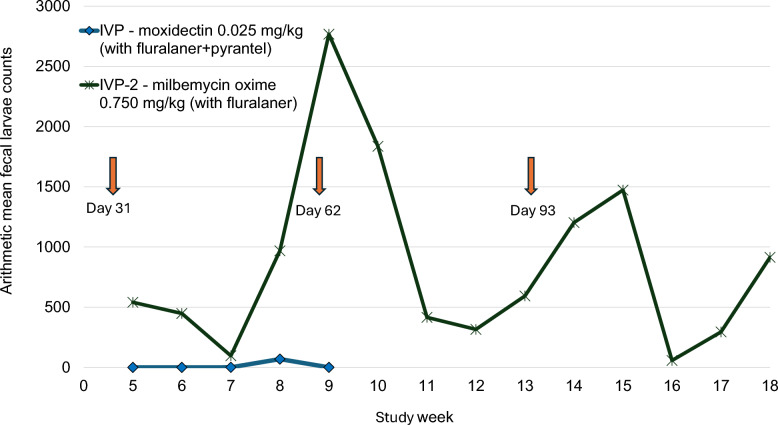
Study 2: weekly arithmetic mean faecal larval counts of dogs treated with a combination of either moxidectin, fluralaner and pyrantel (IVP), once on day 0, or milbemycin oxime with fluralaner (IVP-2) on days 0, 31, 62 or 93 (arrows indicate days of last three IVP-2 treatments)

In the collection period from day 28 to day 62, the geometric (arithmetic) means of the individuals 90th percentile of faecal L1 count of the IVP group was 2.3 (0.4) and significantly different from the CG mean of 13,604.1 (21,585.5) (*F*_1–17_ = 778.84, *P* < 0.0001). In the IVP-2 group (days 93–124), the means of the individuals 90th percentile of faecal L1 count of 95.9 (1,695.8) was significantly different from the CG mean of 52,962.0 (59,531.4) (*F*_1–14_ = 24.91, *P* = 0.0002). Daily arithmetic mean faecal L1 counts in the CG ranged from 905.3 on day 34 to 119,825.7 on day 111. Percentage reductions in geometric (arithmetic) means based on the individuals 90th percentile of faecal larvae counts were 100.0% (100.0%) in the IVP (moxidectin) group, and 99.8% (97.2%) in the IVP-2 (milbemycin oxime) group. In the IVP group, a sufficient amount of faeces (10 g ± 2) for analysis was obtained from at least eight dogs on 19 collection days. No larvae were detected on all but five of those samplings, in four samplings mean counts were either 0.1 or 0.3, while the fifth count (on day 51) was 205.8. In the IVP-2 group, mean faecal larval counts at all 42 collections (samples from at least eight dogs, days 27 to 121) ranged from 2.5 to 6,508. In this group, weekly arithmetic mean faecal larval counts declined soon after each treatment, but then gradually increased with time after treatments (Fig. 2).

### Respiratory tract findings

From thoracic CT, approximately 4 weeks after the *A. vasorum* inoculation on day − 28, lung severity scores had become moderate in all dogs, and bilateral incipient lung pathology was observed with increased attenuation of the lung parenchyma. At subsequent scans, all CG dogs had dilatation of pulmonary arteries, periarterial interstitial infiltrates and filling defects, and bronchial wall thickening with multiple lung lobes affected bilaterally. Lung severity scores were severe in all later scans of all dogs in this group. At the final scan of the dogs of the IVP group, compared with findings of the week prior to treatment, six dogs remained affected bilaterally in multiple lung lobes, two were affected unilaterally in multiple lobes, and in two dogs the pathological changes were no longer present. Mean lung severity scores in the IVP group had improved to normal in two dogs, were mild in seven, and moderate in one dog. Scores in this group at the final scan were significantly different (*P* = 0.0001) from CG scores. Between days 54 and 61, lung severity scores were moderate to severe in all dogs of the IVP-2 group. For the final scans of this group (days 117–124), four dogs had improved to normal, three were scored as mild and three were moderate. Final scan scores of the IVP-2 group differed significantly (*P* = 0.0004) from those of the CG.

There were no overt signs of respiratory involvement at any time in the IVP group. Mild respiratory signs were present in some CG dogs and in one dog of the IVP-2 group. Respiratory frequency in each group was similar until day 7, followed by a marked increase in the CG and a slight increase in the IVP-2 group (Additional file 2; Table S1). The respiratory frequency remained higher in the CG group than in the IVP and IVP-2 groups throughout the remainder of the study. Other respiratory observations (sounds, quality) were normal or mild in all groups.

### Serology (Fig. 3)

**Fig. 3:**
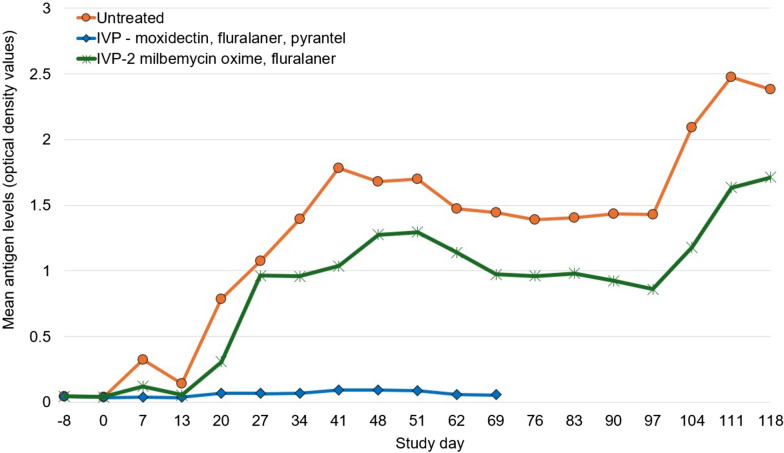
Study 2: mean antigen levels in dogs left untreated, treated once with the investigational veterinary product (IVP) (on day 0) or four times with the IVP-2 (on days 0, 31, 62 and 93)

ELISA results confirmed the presence of circulating antigens of *A. vasorum* in both the CG and IVP-2 groups (Additional file 3; Table S2). While larval counts indicate that few adult worms matured in some dogs in the IVP group, the numbers appear to have been too low for ELISA antigen detection (Fig. 3).

### Studies 3 and 4—efficacy against established *A. vasorum* infections (Table 3)

**Table 3:** Weekly mean faecal counts of first-stage larvae of *Angiostrongylus vasorum* excreted from dogs from studies 3 and 4

Study week	Geometric (arithmetic) mean maximum faecal larvae counts		GM (AM) Reduction (%)	Statistical analysis*	
	IVP	CG		*F*-statistic	*P*-value
Study 3 (8 dogs per group)					
1	156.0 (979.6)	122.6 (333.4)	0.0 (0.0)	0.07	0.8041
2	128.5 (923.0)	207.6 (630.3)	38.1 (0.0)	0.24	0.6372
3	0.1 (0.1)	1107.7 (1638.6)	100.0 (100.0)	394.31	< 0.0001
4	0.0 (0.0)	555.4 (1499.9)	100.0 (100.0)	60.07	0.0001
Study 4 (10 dogs per group)					
1	2687.0 (4713.8)	4762.9 (7550.9)	43.6 (37.6)	1.27	0.2751
2	2766.0 (4857.1)	6640.6 (9022.0)	58.4 (46.2)	2.19	0.1575
3	0.6 (1.7)	6823.3 (11,814.0)	100.0 (100.0)	375.54	< 0.0001
4	0.0 (0.0)	14,104.3 (18,183.0)	100.0 (100.0)	1229.52	< 0.0001

^*^Degrees of freedom (DF) of the statistical tests were 1 (numerator DF) and 17 (denominator DF) in each post-treatment week; CG: control group (mock- or sham-treated); IVP: investigational veterinary product—10 mg/kg fluralaner, 0.025 mg/kg moxidectin, 5 mg/kg pyrantel

Adequacy of infection was confirmed by the recovery of L1 from the faeces of all study dogs before treatment on day 0, and from at least six CG dogs at all subsequent samplings (L1 were recovered from all CG dogs at all samplings). In both studies, differences between groups in geometric mean L1 counts were statistically significant at 3 and 4 weeks post-treatment (*P* < 0.0001) (Table 3). No larvae were detected in samples collected during the final study week from any IVP-treated dog, indicating 100% efficacy in treatment of adult *A. vasorum* infections.

Prior to larval inoculation in Study 3, mean respiratory frequency in both groups was 26.5 breaths per minute. Following inoculation and prior to treatment, maximum mean respiratory frequency in each group had increased by day − 3 to approximately 54 breaths per minute. From days 1–28, the arithmetic mean respiratory frequency in the IVP-treated group ranged from 36.0 on day 28 to 48.0 breaths per minute on day 24, in contrast with the CG in which the respiratory frequency ranged from 48.5 on day 1 to 66.0 on day 17 (Additional file 2, Table S1. Respiratory rates of IVP, IVP-2 and control groups across four studies). In Study 4, overall respiratory rates were lower than in Study 3, although the patterns of increased respiratory frequency post-inoculation and reduction in respiratory rate post-treatment in the treated group were similar in both studies.

The intensity of respiratory sound was scored 0 in all but one IVP-treated dog in Study 3. That dog had a slight intensity and deepened normal respiratory sound recorded on one occasion, while slight intensity and deepened normal respiratory sounds were observed on 14 occasions in CG dogs after day 0. Two instances of abdominal involvement were observed in the CG and one of cough/retch (on day − 3). From day 1 to day 28, five counts of panting were observed in the CG, while no dogs of the IVP group were affected. In Study 4, there were no observations of panting or abdominal involvement, while intensity of respiratory sound was absent or mild in all study dogs. The quality of sound was normal in most CG dogs, deepened normal on two occasions, or deepened normal with wheeze on one occasion.

**Fig. 4:**
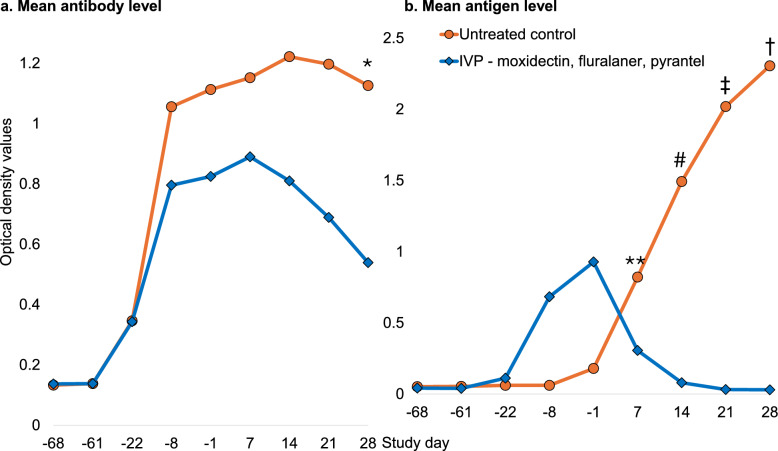
Study 3: mean **a**. antigen, and **b**. antibody levels in treated and control groups. *Angiostrongylus vasorum* third-stage larvae inoculated on day − 60. Treatment with the investigational veterinary product (IVP) on day 0. Statistically significant between-group differences: *F_1–42_ = 4.01, *P* = 0.0375; ^†^*F*_1–13_ = 42.58, *P* < 0001; ^‡^*F*_1–13_ = 36.75, *P* < 0001; ^#^*F*_1–13_ = 28.20, *P* = 0001; ^**^F_1–13_ = 11.67, *P* = 0046

In Study 3, mean antibody levels increased in both groups between days − 61 and − 8 (Fig. 4a). In the IVP group, levels progressively declined from day 14 to 28 when the difference from the CG was statistically significant (*F*_1–42_ = 4.01, *P* = 0.0375). However, there was already a small difference between groups of approximately 0.2 OD on SD-8 to SD 7. Mean antigen levels of both groups in Study 3 were at or close to baseline until a spike in the IVP group on days − 8 and − 1 (Fig. 4b). Following treatment on day 0, levels in this group declined, returning to baseline levels between days 14 and 21, while CG levels continued to increase over the same period. Arithmetic mean antigen levels in the IVP group were significantly different from CG levels on days 7, 14, 21 and 28 (*P* = 0.0046, 0.0001, < 0.0001 and < 0.0001, respectively) (Fig. 4b). In Study 4, the patterns of mean antigen levels in each group were consistent with those in Study 3, with a decline in antigen levels in the IVP group evident after day 7. However, antibody levels between groups do not differ significantly (Additional file 4, Table S3; Additional file 5, Fig S2).

### Adverse events

With the exceptions noted below, adverse events (AE) observed across the studies were generally mild, mostly respiratory signs, and mainly in the untreated control groups. Those events could be attributed to the experimental infections with *A. vasorum*. Two dogs vomited soon after IVP treatment, attributed to the administration. In Study 2, non-serious AEs, manifesting as gastrointestinal disturbance, occurred in most dogs in both treated and untreated groups.

Across the four studies, the only unexpected changes in body weights occurred in Study 2, when three CG dogs were withdrawn for welfare reasons due to weight loss and inappetence, attributable to the experimental infections. An additional dog in this group was withdrawn after developing coagulopathy after the CT. All dogs recovered after treatment, and no other serious AEs occurred in the six dogs remaining in this group.

## Discussion

### Efficacy measured by adult and larval *A. vasorum* counts

The inoculation procedure with L3 was successful in establishing adult *A. vasorum* infections in all four studies. Study 1 (dose determination) showed the appropriate minimum moxidectin dose to be 0.025 mg/kg (combined with 10 mg/kg fluralaner and 5 mg/kg of pyrantel); a single administration resulted in a geometric mean reduction in adult worm counts of 99.0%. The 100% reduction in faecal larval excretion in Study 2 provided confirmation that the selected dose was effective in preventing angiostrongylosis.

Studies 3 and 4 showed that the selected 0.025 mg/kg moxidectin dose was also successful in treating established *A. vasorum* infections. Efficacy was confirmed by the almost total absence of L1 from repeated post-treatment faecal larvae counts, and by reductions in antigen and antibody levels. All four studies reported here align with an earlier publication on the efficacy of a single treatment with a combination product of moxidectin, minimum dose 0.024 mg/kg, with sarolaner and pyrantel [15]. As described in that report, at necropsy, geometric mean adult *A. vasorum* counts were reduced by 94.7% compared to placebo, and mean larval counts were reduced by 100%. In two dose confirmation studies in that report, mean faecal *A. vasorum* L1 counts were reduced by 94.0 and 92.9%, respectively. Relative to the current studies, the lower efficacy reported in those studies is more likely related to different protocols (different canine populations and source of *A. vasorum* L3) than to the slightly lower minimum dose of moxidectin that was adopted.

### Respiratory assessments

It is well established that canine infections with *A. vasorum* are often subclinical. This was generally the case in the four studies included in this report, as most respiratory signs were only detected by focused, protocol-required examinations. However, if left untreated, the incipient pathological pulmonary changes in dogs of the CG, shown in the CT findings of Study 2, would have had the potential to progress, and cause severe and potentially fatal clinical disease. In the IVP group, the pathological changes in the lung had mostly reversed and some treated dogs appeared to have fully recovered. Consistent with the improvement in pulmonary pathology post-treatment, the respiratory rates of the treated dogs declined following treatment and were lower at almost all assessments than in the corresponding CG (Additional file 2; Table S1). Any respiratory signs (panting, intensity and quality of sound, abdominal involvement) were more commonly observed in the control than in treated groups. The results provide strong evidence that a single IVP treatment has the potential to prevent or reduce the pathological changes that can occur in dogs at risk of exposure to infection with *A. vasorum*.

### Serology

The serology results were consistent across all studies, verifying the presence of *A. vasorum* infections in all control groups. In both preventive efficacy studies, antigen levels in the IVP groups remained at or close to baseline. This indicates the lack of development of adult *A. vasorum*, as the assay detects adult E/S antigen. In those treated groups, antibody levels increased in the immediate pre- and post-treatment periods (in the absence of faecal larvae excretion) and then declined. That finding is consistent with a report in which antibodies were detected between 13 and 21 days post experimental inoculation, prior to expected patency [22]. The increase in antibody levels was considered to be related to the immunogenic effect of fourth-stage larvae. In Studies 3 and 4, the return of antigen levels to baseline provided confirmation of the efficacy of the IVP against established *A. vasorum* infections. In all studies, the flat post-treatment antigen levels confirm both the efficacy of the IVP and the value of this test in detecting infection and in assessing a treatment response. Therefore, post mortem assessment of *A. vasorum* worm burden in heart, lungs and pulmonary vessels can be replaced by a combination of appropriate in vivo diagnostic methods, i.e. faecal larval counts, serology, diagnostic imaging and clinical examinations.

### Moxidectin and milbemycin oxime in preventing angiostrongylosis

The dose of milbemycin oxime used in Study 2 (0.75 mg/kg) (IVP-2) was 50% higher than that reported earlier (0.50 mg/kg) [17]. Even at the higher dose, the much higher and sustained serum antigen levels in the IVP-2 group in Study 2 indicate that more adult worms had become established than in the IVP group. In the latter group, antigen levels remained close to zero throughout the study, suggesting few or no adult worms had successfully matured. The conclusion that a higher number of adult worms were established in the IVP-2 than in the IVP group is supported by the very low shedding of L1 detected in the IVP group, while much higher numbers were counted in the IVP-2 group.

The CT scans and clinical observations indicated that there was less pulmonary pathology developing in the IVP group compared to the IVP-2 group. The larval counts following each IVP-2 administration confirm that adult worms remained viable, while the pattern of mean L1 counts indicates that an inhibiting effect on larval shedding of established worms gradually dissipates with time after the IVP-2 treatment (Fig. 2). This is likely due to a temporary embryostatic effect that occurs when adult worms are not killed by a treatment. Such an effect has been demonstrated for macrocyclic lactones, such as ivermectin, in the control of *Onchocerca volvulus* infections in people, and of *D. immitis* infections in dogs [24, 25]. Inhibition of egg shedding, in the absence of an adulticidal effect, has also been reported following ivermectin and oxfendazole treatment of anthelmintic resistant *Haemonchus contortus* infections in sheep. Similarly, treatment of canine multi-drug resistant *Ancylostoma caninum* infections inhibited egg shedding following treatments with either pyrantel pamoate, fenbendazole or milbemycin oxime [26, 27].

## Conclusions

A multi-modular in vivo diagnostic approach was successfully established for the investigation of preventive and curative efficacy of new drug products against *A. vasorum*. These studies demonstrated that a single treatment with the selected minimum moxidectin dose (0.025 mg/kg) in Bravecto TriUNO is highly effective in the prevention of canine angiostrongylosis, by reduction of the level of infection with immature adult (L5) and adult stages of *Angiostrongylus vasorum*. For established infections, Bravecto TriUNO is highly effective in the treatment of canine infections with *A. vasorum*, and can limit or reverse the pathological damage caused by this parasite. Infections with *A. vasorum* were reduced to a lesser extent by four monthly administrations of a combination of milbemycin oxime and fluralaner.

## Supplementary Information

Additional file 1: Fig S1, Study 1. Respiratory rates of dogs in study groups pre- and post-treatment

Additional file 2: Table S1, Respiratory rates of IVP, IVP-2 and control groups across four studies

Additional file 3: Table S2, Study 2. Mean antigen and antibody titers of study groups infected with *Angiostrongylus vasorum* 3rd-stage larvae on day -28. Treatments administered on day 0.

Additional file 4: Table S3, Study 4. Mean antigen and antibody titers of study groups infected with third-stage larvae of *Angiostrongylus vasorum* on day -56. Treatment was administered on day 0.

Additional file 5: Figure S2a, S2b. Study 4. a. Mean antigen and b. mean antibody levels in treated and control groups. *Angiostrongylus vasorum* third-stage larvae inoculated on day -56. [treatment administered on day 0]).

## Data Availability

All relevant supporting data have been included in the manuscript and supplementary files.

## Abbreviations

CG: Control group
IVP: Investigational veterinary product (moxidectin, fluralaner, pyrantel)
IVP-2: Second investigational veterinary product (milbemycin oxime, fluralaner)
L1: First-stage larvae of *Angiostrongylus vasorum*
L3: Third-stage larvae of *Angiostrongylus vasorum*
VICH: The International Cooperation on Harmonisation of Technical Requirements for Registration of Veterinary Medicinal Products
WAAVP: World Association for the Advancement of Veterinary Parasitology
