# Supplementary material for: Efficacy of a single oral administration of a formulation of fluralaner, moxidectin and pyrantel (BRAVECTO® TriUNO) in dogs for the treatment and prevention of angiostrongylosis
Source: Parasit Vectors. 2026 Jul 24;19:303. doi: 10.1186/s13071-026-07529-4 (PMC13411127; doi:10.1186/s13071-026-07529-4)
Supplement: Supplementary file 1 — Additional file 1: Fig S1, Study 1. Respiratory rates of dogs in study groups pre- and post-treatment [file 13071_2026_7529_MOESM1_ESM.pptx]

## Slide 1
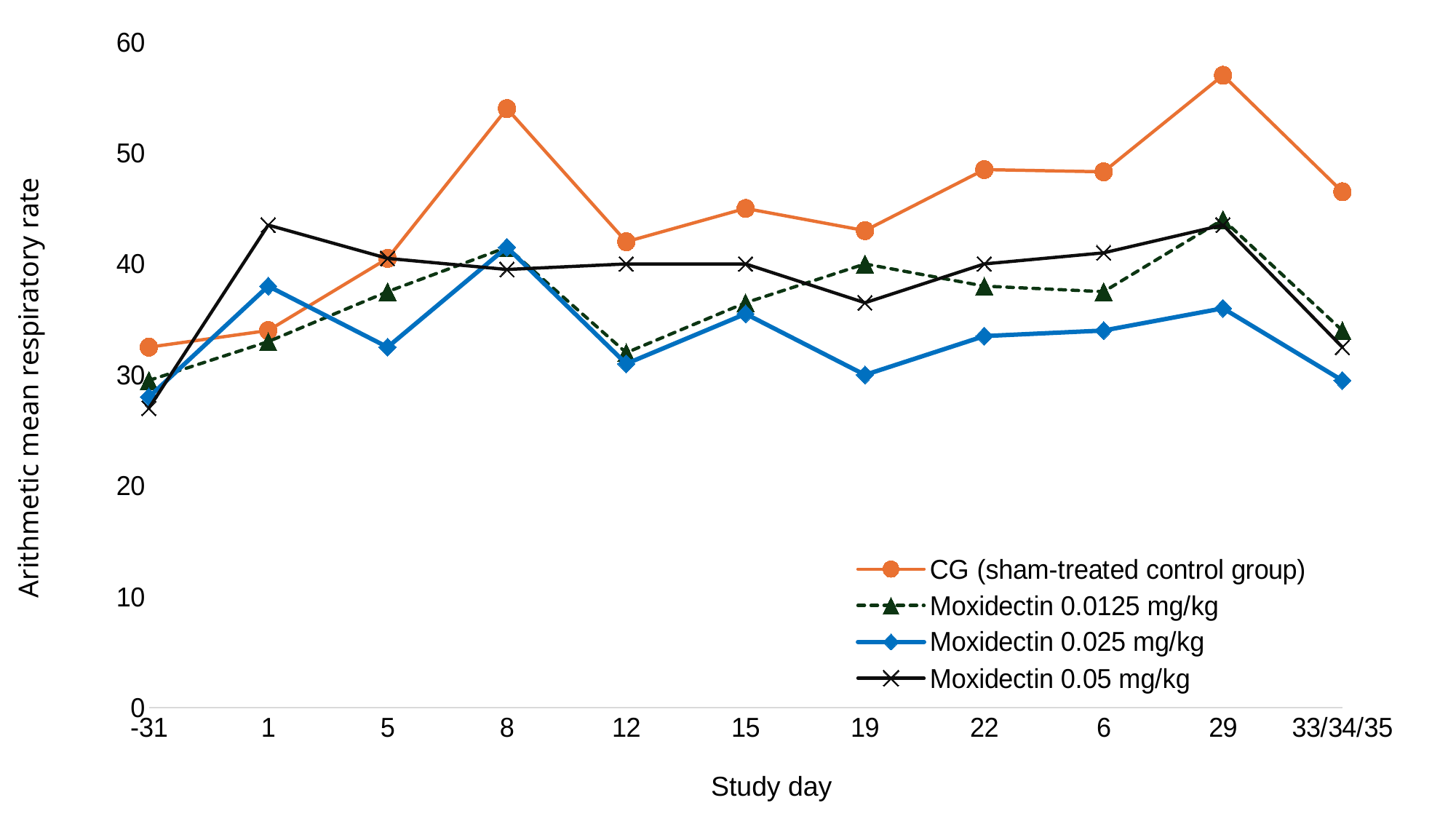

### Chart
| Category | CG (sham-treated control group) | Moxidectin 0.0125 mg/kg | Moxidectin 0.025 mg/kg | Moxidectin 0.05 mg/kg |
|---|---|---|---|---|
| -31 | 32.5 | 29.5 | 28.0 | 27.0 |
| 1 | 34.0 | 33.0 | 38.0 | 43.5 |
| 5 | 40.5 | 37.5 | 32.5 | 40.5 |
| 8 | 54.0 | 41.5 | 41.5 | 39.5 |
| 12 | 42.0 | 32.0 | 31.0 | 40.0 |
| 15 | 45.0 | 36.5 | 35.5 | 40.0 |
| 19 | 43.0 | 40.0 | 30.0 | 36.5 |
| 22 | 48.5 | 38.0 | 33.5 | 40.0 |
| 6 | 48.3 | 37.5 | 34.0 | 41.0 |
| 29 | 57.0 | 44.0 | 36.0 | 43.5 |
| 33/34/35 | 46.5 | 34.0 | 29.5 | 32.5 |Arithmetic mean respiratory rate
Study day
