# Supplementary material for: Efficacy of a single oral administration of a formulation of fluralaner, moxidectin and pyrantel (BRAVECTO® TriUNO) in dogs for the treatment and prevention of angiostrongylosis
Source: Parasit Vectors. 2026 Jul 24;19:303. doi: 10.1186/s13071-026-07529-4 (PMC13411127; doi:10.1186/s13071-026-07529-4)
Supplement: Supplementary file 2 — Additional file 2: Table S1, Respiratory rates of IVP, IVP-2 and control groups across four studies [file 13071_2026_7529_MOESM2_ESM.docx]

Additional file 2, Table S1. Respiratory rates of IVP, IVP-2 and control groups across four studies

|  | Study 1 | | Study 2 | | | Study 3 | | Study 4 | |
| --- | --- | --- | --- | --- | --- | --- | --- | --- | --- |
| Day of study | CG | IVP | CG | IVP-2 | IVP | CG | IVP | CG | IVP |
| -68 – -66 |  |  |  |  |  | 26.5 | 26.5 | 25.2 | 24.8 |
| -60 – -56 |  |  |  |  |  | 31.8^a^ | 38.3^a^ | 26.8^a^ | 28.4^a^ |
| -31 – -28 | 32.5^a^ | 28.0^a^ | 23.6 | 25.2 | 23.6 |  |  |  |  |
| -14 – -7 |  |  |  |  |  | 46.0 | 48.5 | 36.4 | 35.2 |
| -3 – 0 |  |  | 26.0 | 26.4 | 27.2 | 54.5 | 54.3 | 32.0 | 33.6 |
| 0 | Day of treatment, all studies | | | | | | | | |
| 1 | 34.0 | 38.0 | 27.2 | 26.8 | 32.8 | 48.5 | 45.5 | 37.6 | 43.6 |
| 5 – 7 | 40.5 | 32.5 | 50.0 | 27.2 | 30.0 | 48.8 | 41.0 | 40.0 | 31.6 |
| 8 – 10 | 54.0 | 41.5 | 59.6 | 32.9 | 25.3 | 59.0 | 46.5 | 36.8 | 36.0 |
| 12 – 14 | 42.0 | 31.0 | 52.4 | 26.4 | 28.0 | 69.0 | 44.5 | 38.4 | 31.6 |
| 15 – 17 | 45.0 | 35.5 | 46.0 | 41.6 | 23.2 | 66.0 | 37.0 | Study 4 end | |
| 19 – 21 | 43.0 | 30.0 | 45.2 | 26.0 | 32.0 | 49.5 | 38.0 |  |  |
| 22 – 24 | 48.5 | 33.5 | 52.8 | 25.6 | 32.0 | 56.0 | 48.0 |  |  |
| 26 – 28 | 48.3 | 34.0 | 55.1 | 25.2 | 29.2 | 62.0 | 36.0 |  |  |
| 29 – 30 | 57.0 | 36.0 | 56.0 | 39.2 | 26.4 | Study 3 end | |  |  |
| 33 – 37 | 46.5 | 29.5 | 61.2 | 26.4 | 28 |  |  |  |  |
|  | Study 1 end | |  |  |  |  |  |  |  |
| 44 |  |  | 51.2 | 34.8 | 30.0 |  |  |  |  |
| 51 |  |  | 48.6 | 32.4 | 28.8 |  |  |  |  |
| 58 |  |  | 39.4 | 30.8 | 27.2 |  |  |  |  |
| 65 |  |  | 44.0 | 27.2 | 30.0 |  |  |  |  |
| 72 |  |  | 51.4 | 33.0 | Final collection, IVP group | |  |  |  |
| 79 |  |  | 35.4 | 30.8 |  |  |  |  |  |
| 86 |  |  | 44.6 | 28.4 |  |  |  |  |  |
| 93 |  |  | 34.3 | 28.0 |  |  |  |  |  |
| 100 |  |  | 34.9 | 32.8 |  |  |  |  |  |
| 107 |  |  | 37.7 | 29.6 |  |  |  |  |  |
| 114 |  |  | 35.4 | 32.8 |  |  |  |  |  |
| 121 |  |  | 32.7 | 29.2 |  |  |  |  |  |
|  |  |  | Final collection CG and IVP groups | | |  |  |  |  |

^a^Day of challenge with *Angiostrongylus vasorum* third stage larvae (Study 1, day -31; Study 2, day -28; Study 3, day -60; Study 4, day -56)

CG Untreated control group; IVP Investigational veterinary product - fluralaner, moxidectin 0.025 mg/kg, pyrantel; IVP-2 fluralaner, milbemycin oxime
