# Supplementary material for: Efficacy of a single oral administration of a formulation of fluralaner, moxidectin and pyrantel (BRAVECTO® TriUNO) in dogs for the treatment and prevention of angiostrongylosis
Source: Parasit Vectors. 2026 Jul 24;19:303. doi: 10.1186/s13071-026-07529-4 (PMC13411127; doi:10.1186/s13071-026-07529-4)
Supplement: Supplementary file 3 — Additional file 3: Table S2, Study 2. Mean antigen and antibody titers of study groups infected with Angiostrongylus vasorum 3rd-stage larvae on day -28. Treatments administered on day 0. [file 13071_2026_7529_MOESM3_ESM.docx]

Additional file 3, Table S2. Study 2. Mean antigen and antibody titers of study groups infected with *Angiostrongylus vasorum* 3rd-stage larvae on day -28. Treatments administered on day 0

|  | Antigen titers | | | Antibody titers | | |
| --- | --- | --- | --- | --- | --- | --- |
| Study day | Untreated | IVP | IVP-2 | Untreated | IVP | IVP-2 |
| -38 to 30 | 0.020 | 0.021 | 0.021 | 0.098 | 0.098 | 0.098 |
| -22 | 0.034 | 0.032 | 0.032 | 0.153 | 0.151 | 0.148 |
| -150 | 0.032 | 0.031 | 0.031 | 0.284 | 0.303 | 0.366 |
| -8 | 0.042 | 0.043 | 0.046 | 0.314 | 0.336 | 0.403 |
| 0 | 0.038 | 0.040 | 0.039 | 0.164 | 0.172 | 0.154 |
| 7 | 0.325 | 0.041 | 0.122 | 0.213 | 0.195 | 0.192 |
| 13 | 0.141 | 0.041 | 0.055 | 0.797 | 0.442 | 0.712 |
| 20 | 0.785 | 0.066 | 0.308 | 0.767 | 0.487 | 0.843 |
| 27 | 1.076 | 0.066 | 0.863 | 0.931 | 0.534 | 0.949 |
| 34 | 1.394 | 0.067 | 0.959 | 0.833 | 0.516 | 0.907 |
| 41 | 1.783 | 0.092 | 1.038 | 0.607 | 0.758 | 0.747 |
| 48 | 1.682 | 0.092 | 1.275 | 0.886 | 0.721 | 0.828 |
| 51 | 1.701 | 0.090 | 1.296 | 0.835 | 0.790 | 0.849 |
| 62 | 1.475 | 0.061 | 1.142 | 1.028 | 0.386 | 0.950 |
| 69 | 1.444 | 0.058 | 0.974 | 0.984 | 0.359 | 0.871 |
| 76 | 1.389 |  | 0.962 | 0.965 |  | 0.885 |
| 83 | 1.406 |  | 0.981 | 0.997 |  | 0.771 |
| 90 | 1.434 |  | 0.924 | 1.027 |  | 0.905 |
| 97 | 1.428 |  | 0.860 | 0.978 |  | 0.867 |
| 104 | 2.091 |  | 1.180 | 0.921 |  | 0.888 |
| 111 | 2.475 |  | 1.635 | 0.994 |  | 0.806 |
| 118 | 2.383 |  | 1.714 | 1.033 |  | 0.815 |

IVP Investigational veterinary product – fluralaner (10 mg/kg) combined with moxidectin (0.025 mg/kg) and pyrantel (5.0 mg/kg)

IVP-2 fluralaner (10 mg/kg) combined with milbemycin oxime (0.75 mg/kg)

10 dogs in each group, except for the untreated control group in which 3 dogs were removed after day 48.
