# Supplementary material for: Efficacy of a single oral administration of a formulation of fluralaner, moxidectin and pyrantel (BRAVECTO® TriUNO) in dogs for the treatment and prevention of angiostrongylosis
Source: Parasit Vectors. 2026 Jul 24;19:303. doi: 10.1186/s13071-026-07529-4 (PMC13411127; doi:10.1186/s13071-026-07529-4)
Supplement: Supplementary file 4 — Additional file 4: Table S3, Study 4. Mean antigen and antibody titers of study groups infected with third-stage larvae of Angiostrongylus vasorum on day -56. Treatment was administered on day 0. [file 13071_2026_7529_MOESM4_ESM.docx]

Additional file 4. Table S3. Study 4. Mean antigen and antibody titers of study groups infected with third-stage larvae of *Angiostrongylus vasorum* on day -56. Treatment was administered on day 0

|  | Antigen titers | | Antibody titers | |
| --- | --- | --- | --- | --- |
| Study day | Untreated | IVP | Untreated | IVP |
| -66 | 0.059 | 0.066 | 0.117 | 0.117 |
| -1 | 0.650 | 0.062 | 0.689 | 0.642 |
| 7 | 0.827 | 0.549 | 0.708 | 0.701 |
| 14 | 0.878 | 0.307 | 0.759 | 0.670 |
| 21 | 1.079 | 0.185 | 0.889 | 0.752 |
| 28 | 1.043 | 0.108 | 0.805 | 0.588 |

IVP Investigational veterinary product – fluralaner (10 mg/kg) combined with moxidectin (0.025 mg/kg) and pyrantel (5.0 mg/kg)

10 dogs in each group.
