# Supplementary material for: Efficacy of a single oral administration of a formulation of fluralaner, moxidectin and pyrantel (BRAVECTO® TriUNO) in dogs for the treatment and prevention of angiostrongylosis
Source: Parasit Vectors. 2026 Jul 24;19:303. doi: 10.1186/s13071-026-07529-4 (PMC13411127; doi:10.1186/s13071-026-07529-4)
Supplement: Supplementary file 5 — Additional file 5: Figure S2a, S2b. Study 4. a. Mean antigen and b. mean antibody levels in treated and control groups. Angiostrongylus vasorum third-stage larvae inoculated on day -56. [treatment administered on day 0]). [file 13071_2026_7529_MOESM5_ESM.pptx]

## Slide 1
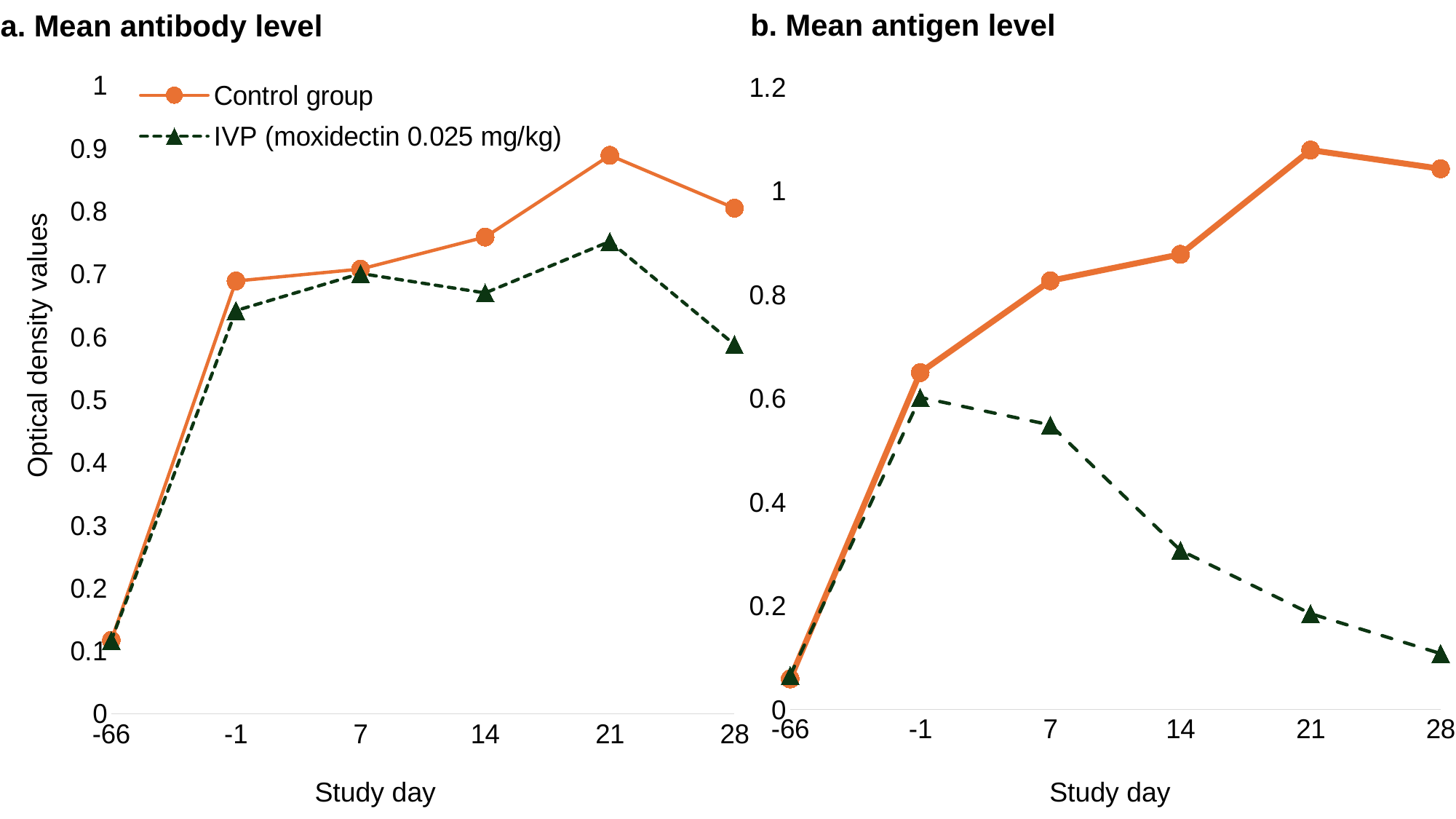

b. Mean antigen level
a. Mean antibody level
### Chart
| Category | Control group | IVP (moxidectin 0.025 mg/kg) |
|---|---|---|
| -66 | 0.117 | 0.117 |
| -1 | 0.689 | 0.642 |
| 7 | 0.708 | 0.701 |
| 14 | 0.759 | 0.67 |
| 21 | 0.889 | 0.752 |
| 28 | 0.805 | 0.588 |
### Chart
| Category | Control group | Moxidecting 0.0125 |
|---|---|---|
| -66 | 0.059 | 0.066 |
| -1 | 0.65 | 0.602 |
| 7 | 0.827 | 0.549 |
| 14 | 0.878 | 0.307 |
| 21 | 1.079 | 0.185 |
| 28 | 1.043 | 0.108 |Optical density values
Study day
Study day
